# Comparison of smith-petersen osteotomy, pedicular subtraction osteotomy, and poly-segmental wedge osteotomy in treating rigid thoracolumbar kyphotic deformity in ankylosing spondylitis a systematic review and meta-analysis

**DOI:** 10.1186/s12893-015-0118-x

**Published:** 2016-01-22

**Authors:** Xumin Hu, Ashish Jung Thapa, Zhaopeng Cai, Peng Wang, Lin Huang, Yong Tang, Jichao Ye, Keng Cheng, Huiyong Shen

**Affiliations:** Department of Spine Surgery, Sun Yat-Sen Memorial Hospital, Sun Yat-Sen University, 107# Yanjiangxi Road, Guangzhou, 510120 P.R. China; Department of Neurosurgery, Sun Yat-Sen Memorial Hospital, Sun Yat-Sen University, Guangzhou, P.R. China

**Keywords:** Smith-petersen osteotomy, Pedicular subtraction osteotomy, Poly-segmental wedge osteotomy, Ankylosing spondylitis, Kyphosis

## Abstract

**Background:**

This study aimed to compare Smith-Petersen osteotomy (SPO), poly-segmental wedge osteotomy (PWO) and pedicular subtraction osteotomy (PSO) in patients with rigid thoracolumbar kyphosis primarily caused by ankylosing spondylitis. The efficiency, efficacy and safety of these three osteotomies have not been compared systematically, and no illness-oriented surgical type selection strategy for the treatment of ankylosing spondylitis related to non-angular kyphosis has been reported.

**Methods:**

The inclusion and exclusion criteria were defined, and 19 electronic databases were searched for eligible studies without language limitations. For the included studies, data extraction, bias analysis, heterogeneity analysis and quantitative analysis were performed to analyze the correction of kyphosiskyphosis and the incidence of complications.

**Results:**

Nine comparative studies that met the standards were included with a total of 539 patients that underwent SPO (*n* = 120), PWO (*n* = 119), or PSO (*n* = 300). The correction of kyphosis by PSO was 8.74° [95 % CI: 0.7-16.78] greater than SPO. The correction of kyphosis by PWO was 13.88° [95 % CI: 9.25-18.51] greater than SPO. For local biomechanical complications, the pooled risk ratio of PWO to PSO was 1.97 [95 % CI: 1.03-3.77]. For blood loss, PSO was 806.42 ml [95 % CI: 591.72-1021.12] greater than SPO and 566.76 ml [95 % CI: 129.80-1003.72] greater than PWO.

**Conclusions:**

To treat rigid thoracolumbar kyphosis, PSO showed higher efficiency and efficacy than SPO, and PWO had a higher efficacy than SPO. The risk of local biomechanical complications was greater in PWO than PSO. Bleeding was more severe in PSO than in SPO or PWO. The incidence of neural complications and systemic complications was similar.

## Background

Ankylosing Spondylitis (AS), a type of chronic disease that involves the axial skeleton, causes severe thoracolumbar kyphotic deformity (TKD). AS makes it difficult for patients to see forward, stand straight and maintain a comfortable posture. Some patients even suffer from dyspnea or other serious complications due to chest compression [1, 2]. Therefore, it is necessary to perform corrective surgeries to help restore spine curvature and visual function [3, 4].

To treat non-angular kyphosis, there are two categories of frequently used surgery. The first, called opening osteotomy (OO), is characterized by “opening” of the anterior column. The Smith-Petersen [5] osteotomy and its modified versions developed by Chapelle [6], Briggs [7], Wilson [8] and Simmons [2] are common choices. SPO only works on one or two segments, so the anterior longitudinal ligament (ALL) and the aorta may be ruptured under highly concentrated stress. In 1982, Zielke [9] increased the segments to three or more. The “elongated SPO” allocates the stress to each segment evenly and is called poly-segmental wedge osteotomy.

The second category is “closing” the posterior column by tri-column osteotomy within one vertebra and is thus termed the closing osteotomy (CO), Thomassen Osteotomy or pedicular subtraction osteotomy; it was first described by Thomassen [10] in 1986. Modified versions of this procedure, such as the “egg-shell” osteotomy and transpedicular bivertebrae wedge osteotomy, are classified as closing osteotomies.

Both opening and closing osteotomies are effective in the treatment of non-angular kyphosis. Several non-comparative clinical trials have attempted to describe the efficacy of kyphosis correction and risk, but many controversies remain. Until now, there was neither a randomized controlled trial (RCT) nor a quantitative meta-analysis on this subject, so the evidence was insufficient to determine which strategy is better. The class of available evidence is not superior to level-3 according to “Oxford 2011 Levels of Evidence” [11], which was established by the OCEBM Levels of Evidence Working Group.

It is important to realize that simply summarizing each individual study without weighting them or equalizing to a baseline as was done in some articles is unacceptable. A meta-analysis of pairwise comparative studies would weight each study by its quality and effectively solve the baseline problem. In order to meet this purpose, raise the level of evidence and highlight some unapparent outcomes by the pile-up effect, the authors of this study intended to perform a meta-analysis to compare Smith-Petersen osteotomy, poly-segmental wedge osteotomy and pedicular subtraction osteotomy from the aspects of efficacy (general correction of kyphosis), efficiency (per level), and safety (complications) in kyphosis correction.

## Methods

### Inclusion and exclusion criteria

Studies meeting all inclusion criteria but none of the exclusion criteria were enrolled. The criteria consisted of 4 parts: type of intervention, type of study, type of participant and type of outcome. A comparison between opening osteotomy and closing osteotomy was acceptable. Controlled studies were eligible. Blindness and allocation concealment were not restricted. Rigid thoracolumbar kyphosis caused by AS or other diseases was eligible. Neither non-rigid nor non-thoracolumbar kyphosis was included. Studies containing patients older than 80 years old or who had an accompanying severe systemic disease like organ failure, malignant tumors or psychosis were excluded, and studies in which patients did not sign consent forms were also excluded. Correction of kyphosis and the incidence of complications were the outcomes of interest. Correction of kyphosis included local correction and that of the whole spine. All information about complications had to be included.

### Search

Without confining the language type, the first two authors filtered the articles by the keywords of osteotomy, osteotomies, ankylosing spondylitis, rigid, fixed, kyphotic, kyphosis, deformity, deformities, thoracic, lumbar, thoracolumbar, sagittal, imbalance, correction, and corrective in databases such as PubMed, Web of Science, Journal Citation Reports (JCR), Derwent Innovations Index, BIOSIS Previews, MEDLINE, Essential Science Indicators (ESI), EMBASE, OVID, ACP Journal Club, Cochrane Central Register of Controlled Trials, Cochrane database of systematic reviews, Cochrane Methodology Register, Database of Abstract of Reviews of Effects, Health Technology Assessment, NHS Economic Evaluation Database, China National Knowledge Infrastructure (CNKI), VIP, and Wanfang Data. The third author was prepared to make a judgement call if any divergence occurred. The date was up to 2015/7/27.

### Data extraction and assessment of study quality

The authors extracted the following information independently and contacted the original authors in case some critical data were found to be lacking: date, study type, patient quantity, gender, age, follow-up period, osteotomy type, operative segments, correction of kyphosis, type and incidence of complications. The second step was the assessment of their quality by means of Cochrane Collaboration’s Tool for Assessing Risk of Bias [12] for RCT, and The Newcastle-Ottawa Scale (NOS) [13] for non-RCT. Subgroup analysis and sensitivity analysis were performed if necessary.

### Heterogeneity and quantitative analysis

Heterogeneity analysis was used to evaluate the differences between studies that were large or small, and the Chi^2^ and I^2^ statistics were used in this step. A *p* value from the Chi square test less than 0.05 and an I square greater than 50 % were considered substantial. Only comparative studies were pooled. The fixed and random mode was used to merge homogenous and heterogeneous data. Subgroup analysis was performed if necessary. The level of the test (α) was set as 0.05. Continuous variables were merged by the general variance-based method. The risk ratio (RR) of dichotomous variables was merged by the Mantel-Haenszel method. All analyses were realigned using Review Manager (Version 5.3) [14] software from the Cochrane Collaboration.

## Results and discussion

### Search results

**Fig. 1:**
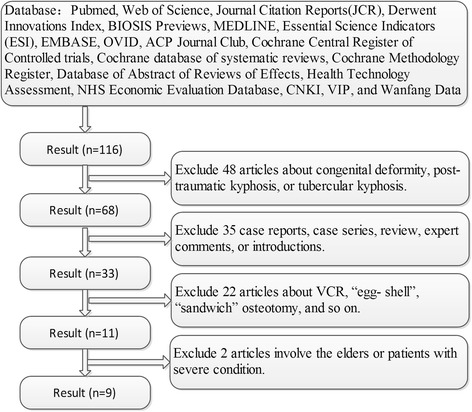
Flowchart of search strategy and results. Information: 9 articles were filtered out from 116 original literatures

The electronic database search and an additional hand search initially yielded 116 citations. All full texts were downloaded from the original database. Of these, 48 did not meet the criteria of participants, 35 were not the correct study type, and 22 did not meet the intervention criteria. Only 9 [15–23] papers were used for the final analysis (Fig. 1). Of these 9 included papers, 8 were retrospective cohort studies and 1 [15] was a conference article. All were published studies, and none were ongoing.

### Data extraction and bias analysis

The 9 studies included 539 patients and 3 types of osteotomies, such as SPO (*n* = 120), PWO (*n* = 119) and PSO (*n* = 300), without the involvement of other modified surgical types, such as the “egg-shell” osteotomy. The cases of SPO consisted of 111 single-level and 9 double-level. However, each PSO was operated in a single level. Four studies [17, 19, 20, 23] reported that PSO was used more often to treat patients with severe stiffness.

The descriptive data of the studies are shown in Table 1. Local biomechanical complications were defined as biomechanical imbalance or instability of the local bone-instrument complex, such as vertebral body translation, pedicle fracture, pedicle screw loosening, instrument breakage, nonunion, and anterior cortex fracture. Neural complications included transient and permanent injury of the radicular and spinal cord. Others, such as superior mesenteric artery syndrome, abdominal compartment syndrome, paralytic ileus, intestinal perforation, dyspnea, pneumonia, cardiac infarction, visual field defect and infection, were classified into systemic complications. The Newcastle-Ottawa Scale was applied to assess whether the biases were too large to combine. The result was encouraging because each text received at least 4 points (median quality). The results are presented in Table 2.

**Table 1:** Descriptive Data of Studies Included

Study	Group	n	Age (range)/year	M:F	Level (NO.)	Correction of lordosis /°	Follow up (range) /Month	Complication (NO.)
Lazennec ^15^	SPO	19	43.5 (32–61)	26:5	T12-L1 (1); L2-L3 (7); L3-L4 (9); L4-L5 (2)	41.1 ± 5.8	-	Dural tear (4); Translation of caudal segment (5); Anterior cortex (1); Unstable fixation (4); Transient neural injury (6); paralysis (1); Narrowing of vertebral canal (1)
	PSO	12			L1 (1); L2 (7); L3 (4)	47.4 ± 4.5		Nonunion (1); Transient neural symptom (3); Secondary displacement (2)
*Qiu* ^20^	PWO	23	36 (25–56)	49:5	T12-L4 (23)	44 ± 12	20 (11–45)	Dural tear (1); Pedicle fractures (1); Insufficient folding (4)
	PSO	31			L2 or L3 (31)	36 ± 19		Dural tear (1); Insufficient folding (2); Transient neural symptom (2)
*Willems* ^27^	PWO	20	46.1 (21–82)	88:22	-	-	12	Dural tear (5); Pedicle fractures (1); Insufficient folding (2); Deep infection (3); Neural damage (1); Instrumentation failure (4);
	PSO	62						Dural tear (12); Pedicle fractures (4); Superficial infection (4); Deep infection (5); Neural damage (6); Instrumentation failure (10); others^a^ (8)
*Cho* ^5^	SPO	16	40.1 ± 11	23:7	Single (7); Double (9)	24.9 ± 10.6	55.2 (24–276)	Dural tear (1); DVT (1); Transient neural symptom (1); Superficial infection (3); Deep infection (1); Sagittal imbalance (4); nonunion (3)
	PWO	14			Triple (9); quadruple (3); quintuple (2)	33.0 ± 9.2		
	PSO	41	54.5 ± 11.7	33:8	-	31.7 ± 9.0	49.4 (24–85.2)	Dural tear (3); DVT (2); Transient neural symptom (3); Superficial infection (1); nonunion (3); others^b^ (3)
*Chang* ^4^	SPO	66	34.8 (17–55)	102:15	L1-L2 (5); L2-L3 (55); L2-L4 (6)	40 ± 14	43.2 (25.2-63.6)	Dural tear (4); Superficial infection (1); Transient neural symptom (3); Nonunion (3); Screw loosing (1); Kyphosis aggravation (2); others^c^ (13)
Study	Group	n	Age (range) /year	M:F	Level (No.)	Correction of lordosis /°	Follow up (range)/Month	Complication (No.)
*Chang* ^4^	PSO	51	34.8 (17–55)	102:15	L2 (47); L3 (4)	38 ± 11	43.2 (25.2-63.6)	Dural tear (3); Superficial infection (1); Transient neural symptom (3); Nonunion (3); Screw loosing (3); Kyphosis aggravation (3); others^c^ (4)
*Zhu* ^29^	PWO	32	35.2 (22–60)	29:3	T11-L4 (8); T12-L4 (21); T12-L5 (3)	39.1 ± 7.8	At least 3	Dural tear (4); Screw loosing (2); Transient neural symptom (1); others^d^ (5)
	PSO	61	37.8 (20–86)	53:8	T12 (2); L1 (11); L2 (30); L3 (18)	37.1 ± 3.8		Dural tear (1); Screw loosing (3); Secondary displacement (2); Fatal Bleeding > 4000 ml (5); Transient neural symptom (4); others^d^ (5)
Arun^1^	SPO	10	54.7 (40–74)	26:5	L3-L4 (10)	19 ± 11.6	60 (24–240)	Dural tear (2); Aorta damage (1); Nerve root injury (1); nonunion (1); Epidural hematoma (1)
	PWO	9			L2-L5 (9)	30 ± 6.2		Dural tear (1); Superficial infection (1); Epidural hematoma (1)
	PSO	12			L3 (12)	38 ± 5.4		Dural tear (1); Nerve root injury (1); Superficial infection (1); Epidural hematoma (1)
Zhu^30^	PWO	19	27 (21–40)	16:3	T11-L4 (4); T12-L4 (14); T12-L5 (1)	38.6 ± 12.8	At least 12	Dural tear (2); Transient neural symptom (1);
	PSO	31	36 (22–54)	26:5	L1 (9); L2 (14);L3 (8)	42.6 ± 15.7		Dural tear (1); Transient neural symptom (3); Neural damage (1)

Note:^a^ indicates pulmonary embolism, blindness, gastrointestinal perforation, or death; ^b^ myocardial infarction or dyspnea; ^c^ indicates pneumonia or enteroplegia. Age (range), mean and range of age of patients; M:F, ratio of male to female; Level, the level of osteotomy operated; NO., the number of osteotomy segment or happening of complication; SPO, Smith-Petersen osteotomy; PWO, poly-segmental Wedge Osteotomy; PSO, pedicle subtraction osteotomy; ^d^ gastrointestinal perforation, enteroplegia, or Syndrome of superior mesenteric artery

**Table 2:** Assessment of studies

Categories	Items	Lazennec^15^	Qiu^20^	Willems^27^	Cho^5^	Chang^4^	Zhu^29^	Arun^1^	Zhu^30^
Selection	Representativeness of the Exposed Cohort	Not described	Not described	Not described	Not described	Not described	Not described	Not described	Not described
	Selection of the Non-Exposed Cohort	The same population	The same population	The same population	The same population	The same population	The same population	The same population	The same population
	Ascertainment of Exposure	Surgical document	Surgical document	Surgical document	Surgical document	Surgical document	Surgical document	Surgical document	Surgical document
	Outcome of Interest Was Not Present at Start of Study	Sure	Sure	Sure	Sure	Sure	Sure	Sure	Sure
Comparability	Comparability of Cases and Control	With confounding factors	With confounding factors	With confounding factors	With confounding factors	With confounding factors	With confounding factors	With confounding factors	With confounding factors
Outcome	Assessment of Outcome	Reliable	Reliable	Reliable	Reliable	Reliable	Reliable	Reliable	Reliable
	Was Follow-Up Long Enough for outcomes to occur?	Not described	Yes	Yes	Yes	Yes	Yes^a^	Yes	Yes
	Loss of Follow Up	Full follow-up	Not described	Full follow-up	Full follow-up	Full follow-up	Full follow-up	Full follow-up	Full follow-up
Score		5	4	6	6	6	5 or 6^b^	6	6

Note: ^a^ Zhu reported at least 3-month follow-up which satisfied the observation of immediate correction of lordosis and neural symptom but not bony union; ^b^ 5 points for assessment of bony union and 6 for lordosis correction. The full mark is 8 points

### Correction of kyphosis

**Fig. 2:**
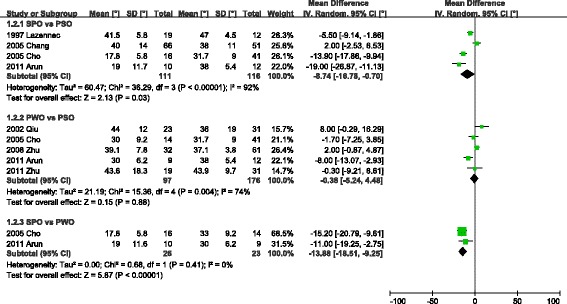
Comparison of three types of osteotomy on kyphosis correction among SPO, PSO and PWO. Information: Both PSO and PWO had bigger correction angle than SPO

The pooled data of kyphosis correction is presented in Fig. 2. In the comparison of SPO and PSO, the former correction of kyphosis ranged from 19° to 41.5° with a mean of 35.2°, and PSO ranged from 31.7° to 48° with a mean of 36.7°. The correction angle of SPO was 8.74° [95 % CI: 0.7-16.78] lower than PSO (I^2^ = 92 %). In the comparison between PWO and PSO, the former ranged from 30° to 44° with a mean of 39.0°, while PSO ranged from 36° to 43.9° with a mean of 36.1°. The correction angle of PWO was similar to PSO (mean difference: 0.38° [95 % CI:–4.48-5.24]. In the comparison of SPO and PWO, the former ranged from 17.8° to 19° with a mean of 18.3°, and PWO ranged from 30° to 33° with a mean of 31.8°. The SPO was 13.88° [95 % CI: 9.25-18.51] lower than PWO (I^2^ = 0).

### Complications

#### Local biomechanical complications

**Fig. 3:**
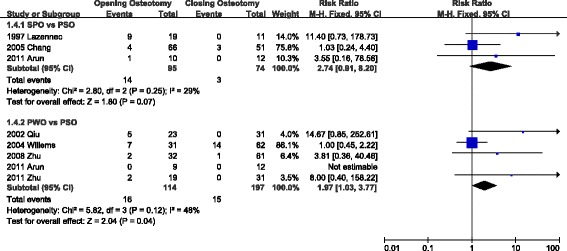
Title: Comparison of local biomechanical complications in SPO, PSO, and PWO; CI. Information: The risk ratio of PWO to PSO was 1.97 and significant

Seven articles reported local biomechanical complications (Fig. 3). The authors excluded Cho’s paper because it contained non-rigid congenital and degenerative kyphosis. In the comparison of SPO and PSO, the RR ranged from 1.03 to 11.40 with a pooled value of 2.74 [95 % CI: 0.91-8.20]. In the comparison of PWO and PSO, the RR ranged from 1.00 to 14.67 with a pooled value of 1.97 [95 % CI: 1.03-3.77] (I^2=^48 %).

#### Blood Loss

**Fig. 4:**
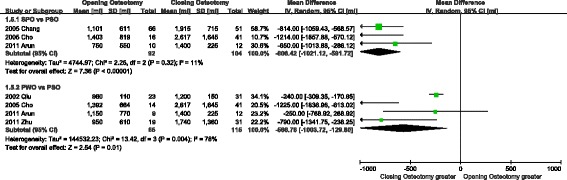
Comparison of blood loss; CI, confidence interval. Information: Both SPO and PWO had fewer blood loss than SPO

Three articles [15, 16, 18] did not record blood loss, or it was recorded improperly. In the comparison of SPO and PSO, the blood loss of the former ranged from 750 ml to 1101 ml with a mean of 1115 ml, and PSO ranged from 1400 ml to 1915 ml with a mean of 2132 ml. The blood loss of PSO was 806.42 ml [95 % CI: 519.72-1021.12] greater than SPO (I^2^ = 11). In the comparison of PWO and PSO, the former ranged from 950 ml to 1392 ml with a mean of 1076 ml, and PSO ranged from 1200 ml to 2617 ml with a mean of 1872 ml. The blood loss of PSO was 566.76 ml [95 % CI: 129.80-1003.72] greater than PWO (I^2^ = 78). Generally speaking, the amount of bleeding presented in Fig. 4 was more severe in closing osteotomy than in opening osteotomy.

### Dural tear, neural complications, and systemic complications

**Fig. 5:**
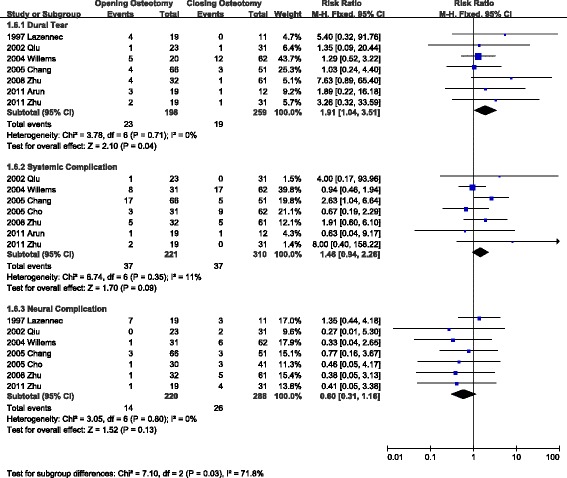
Comparison of dural tear, neural complications, and systemic complications

Seven articles reported the incidence of dural tear, neural complications, and systemic complications. As is shown in Fig. 5, the pooled RR of dural tear was 1.91 [95 % CI: 1.04-3.51], and the p value was less than 0.05 (I^2^ = 0). The RR of systemic complications was 1.46 (not significant, I^2^ = 11 %). The RR of neural complications was 0.6 (not significant, I^2^ = 0). The authors attempted to separate radicular and spinal injuries to perform subgroup analysis but failed due to a shortage of suitable studies.

## Discussion

Four pooled studies used opening osteotomy (SPO or PWO) to treat mild-rigid spine, and closing osteotomy (PSO) to severe-rigid spine, because the anterior column of mild-rigid spine can be bent backwards easily in the process of “opening”, while the stiffness of the severe-rigid spine can only accept “cutting and closing” in a closing osteotomy. This point of view is generally recognized as consensus by most surgeons. In this research, the stiffness of spine was simply divided into three categories based on their degree of stiffness and the surgical type doctors considered appropriate. For the very soft or very rigid kyphosis, doctors were already able to choose the suitable operation easily based on their expertise as mentioned, so the operation selection guidance for those cases was not necessary. However, they usually felt hesitation about the intersectional stiffness between mild and severe since both opening and closing methods seemed good to use, therefore we define this state of stiffness as “median-rigid kyphosis” These cases are actually confusing to surgeons attempting to choose a suitable method.

**Fig. 6:**
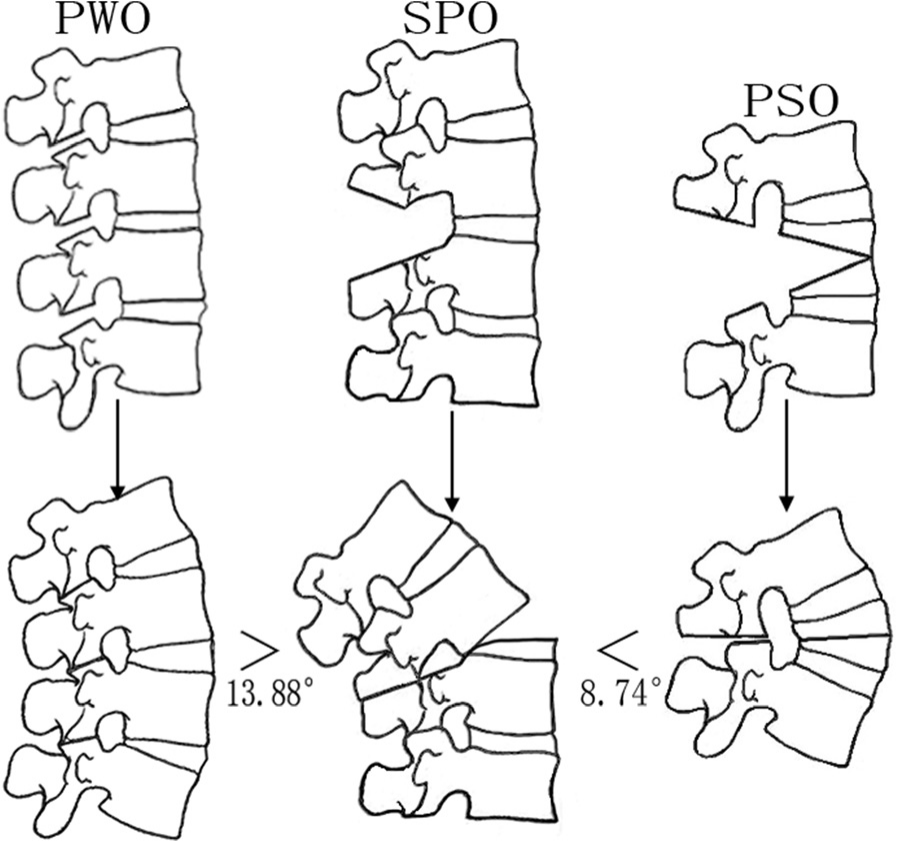
Schematic comparison of Smith-Petersen osteotomy, poly-segmental wedge osteotomy and pedicle subtraction osteotomy for the correction of kyphosis. Information: The correction of kyphosis of PWO was 13.88° greater than SPO. The correction of kyphosis of PSO was 8.74° greater than SPO

The efficiency of kyphosis correction in osteotomy can be defined as the correction of kyphosis per surgical level. As described above, the kyphosis correction of PSO was larger than SPO, so the efficiency of PSO was greater than SPO in treating median-rigid cases. The reasons can be explained from two aspects. First, every PSO was single-level, but 9 out of 120 SPO cases were double-level, so the correction angle of a single-level SPO was smaller if we excluded the double-level cases. Second, even if the severe-rigid cases were included, PSO still had a larger correction angle than SPO. If PSO is used to cure mild-to-median rigidness, the correction angle should not decrease. On the other hand, the correction angle of SPO is difficult to increase during the treatment of severe stiffness. PWO had a larger correction efficacy than SPO because PWO involves more segments. Because PWO wins by the sheer quantity of surgical levels, the outcome is unlikely to change in median-rigid cases (Fig. 6).

The correction angles of some individual cases were quite extreme, such as 52° for SPO [15] and 60° for SPO [2, 24]. As the angle increases, the incidence of complications increases. Wide-angle SPO usually causes lethal bleeding by aortic damage. In all 9 included studies, only Arun [22] reported one death. This reflects the fact that surgeons clearly know of the problem and paid great attention to avoid such a danger. Arun [22] emphasized the importance of a slow and careful operation during the “opening” process. In other words, do not let the “click” sound occur. The sound “click” came from the fracture of bone when spine was pulled backwards rapidly. Chang [19] did not suggest performing SPO on the elderly, advanced AS patients, or patients with arteriosclerosis. Chang [19] and Arun [22] considered SPO to be relatively suitable for L2 and lower levels, and malleable rods were necessary to offer temporary stability in the case of a correction greater than 35°. Because each method has its limitations, seeking a one-step correction in a single level is very dangerous. Bridwell [18] suggested combining one PSO and several PWOs together. SPO and PWO are not essentially different, except for the quantity of levels.

Local biomechanical complications included vertebral body translation, pedicle fraction, pedicle screw loosening, instrument breakage, nonunion, and anterior cortex fracture. They are mostly caused by improper fixation, miss operation or excessive elastic stress. PWO has a higher incidence of local biomechanical complications than PSO because PWO needs to overcome a greater stress of the spine. For mild-rigid cases, PWO would be more difficult in the median-rigid cases. On the contrary, using PSO to treat median-rigid cases should be easier compared to the severe cases reported by the pooled four studies. In other words, the rigidness of the spine affects PWO much more than PSO. Of the fixation materials, the Universal Spine System (USS) was stronger [15, 17] than the slender rod. Royen [25] reported that the instrumentation failure rate in PWO (6.5 %) was higher than PSO (3.8 %), which was close to our findings. Zhu [20] reported that the rate of correction loss in follow-up during opening osteotomy was 6.1° ± 6.7°, while that of the closing osteotomy was 1.3° ± 5.4°. Surgeons [15, 23] found that high anterior column tension, osteoporosis and progression of AS were too difficult and dangerous to perform for opening osteotomy because the implementation of SPO and PWO rely on a powerful pivot (posterior margin of vertebrae) to get the anterior column to open.

Blood loss in PSO was 800 ml greater than in SPO and 550 ml greater than in PWO. The large amount of blood loss in PSO was due to deeply cutting the vertebrae and difficulty in hemostasis. The key steps of PWO and SPO are the resection of the disc and zygapophyseal joint, during which bleeding would not be as many of a problem. In addition, PSO is more frequently used in severe-rigid cases. Thus, the more rigid the bone is, the more fragile the vessel becomes. As such, closing osteotomy should be carefully performed on the elderly and patients with a low tolerance to hemorrhage.

Some non-significant but highly consistent pooled outcomes appeared as well. In the analysis of local biomechanical complications, every individual study consistently presented that the incidence in SPO was greater than PSO. It is very likely that a meaningful outcome would be found if more articles were included or if each included study had a smaller standard deviation (high uniformity). We considered the incidence of local biomechanical complications in SPO to be higher than in PSO in median-rigid cases.

Cho [18] found better improvement in the sagittal vertical axis (SVA) in PSO than in PWO after the same angle of correction because PSO swings the upside vertebral column backward, and PWO twists it instead. He recommended using PSO to treat a SVA greater than 12 cm. Daubs [26] thought that 10 cm was more reasonable.

The incidence of systemic complications was nearly the same in each individual study. The causes, such as inflammatory status of AS, medical co-morbidity, and perioperative management, might be complicated. Multifactor regression analysis and the inclusion of more related articles are needed for further analysis. Dura mater tearing is caused by vertebral translation [25] and careless clamping off of the calcified ligamentum flavum.

The spinal cord and nerve root are likely to be injured during the transformation of kyphosis. Royen [25] and Lazennec [15] believed that PSO did less harm to the nerve root because the previous steps, laminoplasty and intervertebroplasty, generally broadened the nerve root canal. However, PSO is not a good option for the spinal cord. A PSO greater than 40° would increase the probability of spinal cord compression by shortening the posterior column [27]. Chang [19] did not encounter such problems even during PSOs of greater than 45°. He thought careful manipulation and the high tolerance of the medullary cone to compression were helpful. By calculating the height of a normal lumbar vertebral body, some surgeons consider that a PSO greater than 35° [19, 28, 29] was difficult to achieve theoretically in one level due to greater nerve injury risk. The angle approaches our result (36°).

## Conclusions

The “median-rigid” was not an exact quantified degree, but just the remaining cases without prominent features of stiffness that could not be recognized by doctors in decision making. The authors mainly discussed the operation choose for these cases. The authors closely combined surgeon’s specialized knowledge with evidence-based data in order to help make right operation decisions. Median-rigid cases can be treated by either opening or closing osteotomy; PSO is more effective and efficient than SPO, and PWO is more efficient than SPO. PSO is suitable for high-degree kyphosis. Its advantages are little harm to the aorta, small demand on bone density, and low risk of instrumentation failure. However, substantial blood loss and the complicated surgical technique are two factors doctors should seriously consider. Comparing with SPO, PWO is proper for large angle kyphosis, but doctors must overcome such high risk of instrumentation failure. SPO is really good for small angle. Careful work is necessary to avoid aorta damage and middle column fracture. There are some limitations to this study. I^2^ value more than 50 % is customarily considered to be high heterogeneity between studies. The I^2^ value in the correction angle comparing PSO vs SPO is 92 %. By now it is inappropriate to remove any articles no matter from professional judgement or the weight each possesses, because too few are included. To reduce the confounding effect of high heterogeneity, we used random mode which was supposed to give a very conservative result that not easy to make sense. The correction angle of SPO and PSO was regarded to be different in case the result under random mode still showed significance, and that was the fact. However, low heterogeneity degrade the level of evidence. To recognize and eliminate the bias completely, an RCT was needed. With the publication and enrollment of more new studies, these conclusions will become more convincing.

## Abbreviations

ALL: anterior longitudinal ligament
CO: closing osteotomy
NOS: Newcastle-Ottawa scale
OO: opening osteotomy
PSO: pedicular subtraction osteotomy
PWO: poly-segmental wedge osteotomy
RCT: randomized controlled trial
RR: risk ratio
SPO: smith-petersen osteotomy
TKD: thoracolumbar kyphotic deformity
